# Photoacoustic Energy Sensor for Nanosecond Optical Pulse Measurement

**DOI:** 10.3390/s18113879

**Published:** 2018-11-11

**Authors:** Pil Gyu Sang, Junseok Heo, Hui Joon Park, Hyoung Won Baac

**Affiliations:** 1 Department of Electrical and Computer Engineering, Sungkyunkwan University, Suwon 16419, Korea; pgsang@skku.edu; 2 Department of Electrical and Computer Engineering, Ajou University, Suwon 16499, Korea; jsheo@ajou.ac.kr; 3 Department of Energy Systems Research, Ajou University, Suwon 16499, Korea

**Keywords:** ultrasound sensor, photoacoustic sensor, optical energy meter, CNT-PDMS, nanosecond optical pulse

## Abstract

We demonstrate a photoacoustic sensor capable of measuring high-energy nanosecond optical pulses in terms of temporal width and energy fluence per pulse. This was achieved by using a hybrid combination of a carbon nanotube-polydimethylsiloxane (CNT-PDMS)-based photoacoustic transmitter (i.e., light-to-sound converter) and a piezoelectric receiver (i.e., sound detector). In this photoacoustic energy sensor (PES), input pulsed optical energy is heavily absorbed by the CNT-PDMS composite film and then efficiently converted into an ultrasonic output. The output ultrasonic pulse is then measured and analyzed to retrieve the input optical characteristics. We quantitatively compared the PES performance with that of a commercial thermal energy meter. Due to the efficient energy transduction and sensing mechanism of the hybrid structure, the minimum-measurable pulsed optical energy was significantly lowered, ~157 nJ/cm^2^, corresponding to 1/760 of the reference pyroelectric detector. Moreover, despite the limited acoustic frequency bandwidth of the piezoelectric receiver, laser pulse widths over a range of 6–130 ns could be measured with a linear relationship to the ultrasound pulse width of 22–153 ns. As CNT has a wide electromagnetic absorption spectrum, the proposed pulsed sensor system can be extensively applied to high-energy pulse measurement over visible through terahertz spectral ranges.

## 1. Introduction

Nanocomposite thin films, consisting of carbon nanotube (CNT) and polydimethylsiloxane (PDMS), have been widely used for highly efficient photoacoustic energy conversion [1,2,3]. Under pulsed laser irradiation with a short temporal width, these thin-film transmitters can produce pressure pulses with a broad frequency bandwidth over tens of MHz and a high peak amplitude on the order of MPa. The nanocomposite structure exhibits not just efficient energy conversion but also strong robustness allowing high-energy optical absorption (>100 mJ/cm^2^) without laser-induced ablation [4]. Due to these advantages, CNT-PDMS transmitters have been utilized for all-optical ultrasound transducers [5,6], fiber-optic ultrasound generators [7], energy conversion lenses for laser-generated focused ultrasound (LGFU) [8,9] and transduction layers to generate micro-bubbles and shear forces for safe harvesting of cultured cells [10].

Nanoscale light absorbers can be instantaneously heated by pulsed laser irradiation with short temporal widths (e.g., <10 ns). Due to low specific heat capacity, the optically deposited thermal energy is then rapidly transferred to the surrounding medium. This mechanism has been utilized for photothermal therapy [11] and photoacoustic generation of ultrasound [12]. Moreover, the latter has enabled development of photoacoustic transmitters, for example, using CNT-PDMS composite films [1,3]. In the CNT-PDMS composite structure, PDMS receives the thermal energy from the nanoscale heater and then generates acoustic pressure via volume expansion [1,2,3,4,8]. In this photoacoustic process, the incoming laser beam characteristics are fully reflected into the acoustic output in terms of temporal pulse width, peak magnitude and waveform [1].

Particularly, CNTs among various nanoscale elements have a broad absorption spectrum covering a range from visible light through terahertz (THz) electromagnetic waves [13]. Recently, it has been shown that THz pulse radiation can even be absorbed by CNT-PDMS composite to generate short pressure pulses [14]. These pulses were acoustically detected by a polymer microring-based optical resonator. Although this combined system demonstrated real-time detection of THz pulses, its usability was limited to the THz range. Moreover, most of the incoming THz wave field was spatially wasted in the sensing process because a microscale ultrasound detector has a tiny area with only 0.075% of the spatial extent of an incident THz field with a diameter of 0.8 mm. Such a small sensing area is not necessary as incident THz or visible range beams are normally mm to cm in scale.

We demonstrate a photoacoustic energy sensor (PES) efficiently designed to measure nanosecond laser pulses in terms of pulsed energy fluence and temporal width. The PES consists of two parts: (1) a planar CNT-PDMS transmitter working as the converting element transforming an incident pulsed laser beam into an ultrasonic output and (2) a piezoelectric receiver to detect the photoacoustic output produced from the transmitter. The PES was used to measure pulsed laser energy fluences over a wide dynamic range of 55 dB, providing time-domain waveforms in real time. Such instantaneous measurement of waveforms is not available from typical energy meters used for pulsed laser measurement. Moreover, for high-energy measurements, we utilized the extraordinary mechanical robustness of the CNT-PDMS composite film against pulsed laser irradiation [4], which other optical transmitters such as metallic coatings or patterned nanostructures do not possess [15]. This enabled the PES to reproducibly measure an optical fluence as high as >100 mJ/cm^2^ without any damage. Simultaneously, the PES exhibited significantly high sensitivity in detecting an optical energy fluence as low as 157 nJ/cm^2^. The high sensitivity is a benefit of the wide sensing area of the piezoelectric receiver, which can cover the full spatial extent of the photoacoustic field generated from the CNT-PDMS transmitter. Particularly, PES operation can be extended to accommodate acoustic receivers with various geometrical shapes, sensitivity and frequency spectral responses. Here, we utilize two types of piezoelectric receivers to fabricate PES devices: one with a wide aperture seeking an enhanced sensitivity and the other with a broadband frequency response for pulse width characterization. We compare the performance of our PES with that of a commercially available pyroelectric sensor. We also discuss our PES configuration by comparison with the system previously reported for measurement of THz radiation.

## 2. Materials and Methods

CNT-PDMS composite films have been previously used as photoacoustic transmitters to generate high-amplitude and high-frequency ultrasound [1,8]. In this composite transmitter, CNT elements can absorb nanosecond pulsed laser beams, working as instantaneous optical heaters. Then, most of the thermal energy optically deposited within the CNT can be transferred rapidly to the surrounding PDMS (>90% during optical pulse width). As the PDMS with a high coefficient of thermal expansion (0.92 × 10^−3^ K^−1^) is configured as the surrounding matrix, the composite transmitter enables thermoacoustic generation of high-amplitude pulsed ultrasound [2,4].

**Figure 1:**
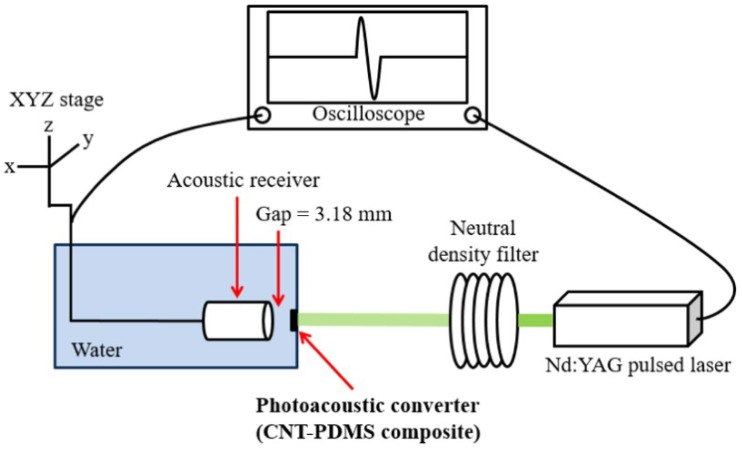
An experimental setup for nanosecond pulsed laser measurement using a combination of a carbon nanotube-polydimethylsiloxane (CNT-PDMS) transmitter and an acoustic receiver. Two acoustic receivers were used alternately for characterization: a wide-aperture piezoelectric receiver (25-mm diameter) for high-sensitivity measurements and a broadband needle hydrophone (20-MHz bandwidth) for pulse width measurements.

Here, for nanosecond optical pulse measurement, we used an Nd:YAG laser beam (532-nm wavelength, 6-ns pulse width, 10-Hz pulse repetition rate, 8-mm beam diameter; Litron Lasers, Rugby, UK) as an input to our sensor system (Figure 1). The laser beam was incident into the glass substrate side and then absorbed by the CNT-PDMS film (<20 μm thickness). The CNT-PDMS transmitter carries out photoacoustic conversion, which generates output ultrasound pulses transmitted via an acoustic impedance-matching medium (deionized water) to a piezoelectric receiver.

**Figure 2:**
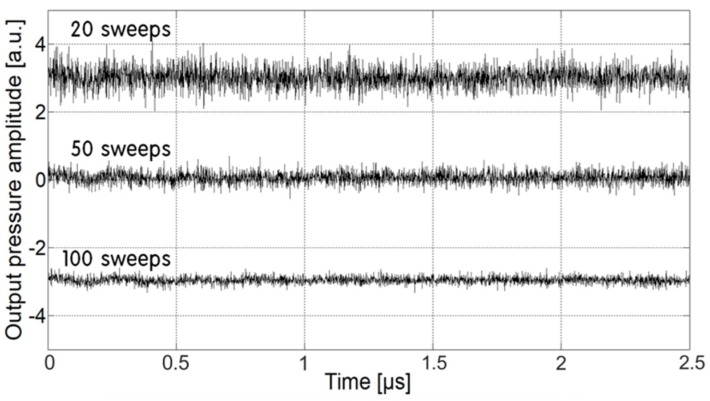
Background noise of the photoacoustic energy sensor (PES) system without pulsed laser irradiation. The noise level can be decreased by taking the average of greater numbers of sweeps.

For high-sensitivity measurements, we first used a PES configuration using the CNT-PDMS transmitter and an acoustic receiver with a 3-MHz operating frequency and a wide diameter of 25 mm (Valpey Fisher E1654, Hopkinton, MA, USA). In Figure 2, the background noise of the PES is shown with averaged waveforms. The noise could be reduced by increasing the number of sweeps and taking an average. The root-mean-square noise amplitude was 21.8, 15.5 and 11.6 μV for 20, 50 and 100 sweeps, respectively. We used 50 sweeps for all of the following measurements. The PES performance was compared with that of a commercial optical energy meter (Thorlabs, Newton, NJ, USA) relying on pyroelectric measurements. This energy meter exhibited a similar sensing dimension (diameter: 24 mm) and a high damage threshold of 800 mJ/cm^2^ for pulse widths <100 ns.

Then, we prepared a second configuration for pulse-width measurements in which the acoustic receiver was replaced with a needle-type hydrophone coated with polyvinylidene fluoride (PVDF) that has a wide bandwidth up to 20-MHz and a diameter of 1 mm (Precision Acoustics, Dorset, UK). The PES was irradiated by the pulsed laser beam with various temporal widths from 5.6 to 128 ns. In order to check the reliability of the PES measurements, optical pulse widths were separately confirmed by using a high-speed photodetector with a 125-MHz bandwidth (Newport, 1811-FC, Irvine, CA, USA). We note that the photodetector could be only used to provide reference pulse widths for comparison but it was not suitable for high-energy pulse measurement due to a very low laser damage threshold (<1 mJ/cm^2^).

## 3. Results and Discussion

For CNT-PDMS composite transmitters, a photoacoustic output pressure can be represented approximately by using the following equation [2]:

(1) $$P=\Gamma \cdot A\cdot \frac{F}{c\tau +1/\alpha }$$

where *P*, *Γ*, *A*, *F*, *c*, *τ* and *α* are the pressure amplitude, the dimensionless Grüneisen coefficient, the optical absorption, the incident optical fluence, the sound speed, the laser pulse width and the depth of optical absorption, respectively. This shows the linear dependence between the incident laser energy fluence and the output pressure of the transmitter. For a given laser pulse width (*τ*) and a CNT film thickness (*α*), the CNT-PDMS transmitter can generate high-amplitude pressure due to the high Grüneisen coefficient (*Γ* ~0.72) of PDMS and its high optical absorption (*A* ~1). In our PES system, such high-amplitude pressure from the CNT-PDMS is measured by using an additional acoustic receiver.

**Figure 3:**
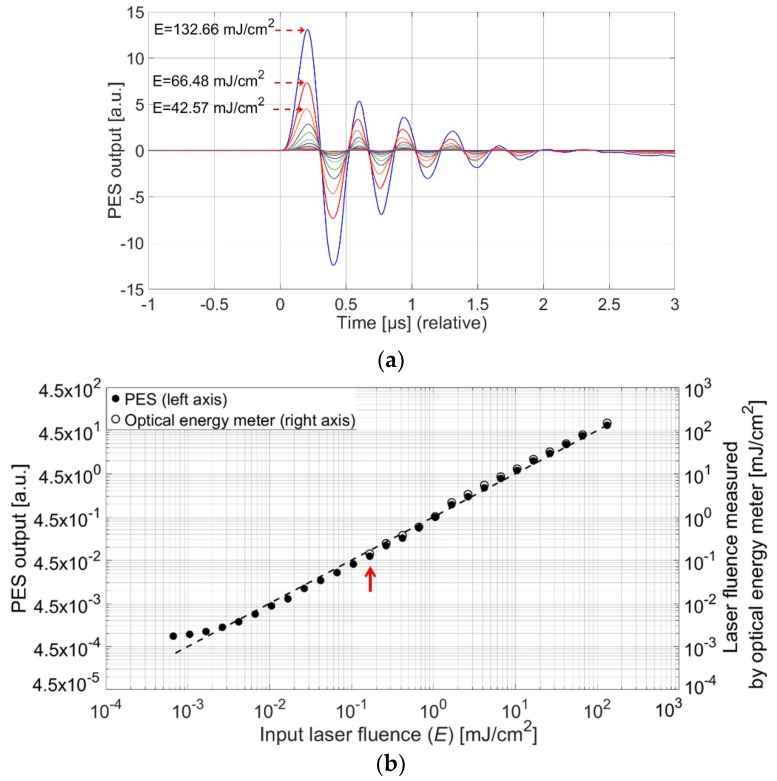
(**a**) Ultrasound waveforms measured by PES for an incident laser energy fluence of 132.66 mJ/cm^2^–156.96 nJ/cm^2^. (**b**) Comparison of the pulse laser energy fluence measured by PES (filled circle; left axis) with the reference measured by the optical energy meter (empty circle; right axis). Here, the dotted line indicates when an input optical fluence from the Nd:YAG laser is the same as the output fluence detected by the optical energy meter. Note that the lower range of fluence (below the arrow) could not be measured by the reference detector.

Figure 3a shows the waveforms measured using the PES with the CNT-PDMS film and the wide-aperture receiver. Although a single Gaussian photoacoustic pulse was generated initially from the surface of the CNT-PDMS transmitter [1], the output of the PES was observed with a ringing tail in the waveform. This is due to the limited frequency bandwidth of the piezoelectric receiver. However, the input laser fluence shows linear relationships with the output peak pressure amplitude and the acoustic vibration in time. As the acoustic vibration results solely from the optical input, the entire acoustic vibration can be integrated in time to quantify the laser energy fluence.

In Figure 3b, the PES output was obtained by integrating the acoustic vibration in time. In this characterization, the pulsed laser energy fluence at the exit of Nd:YAG laser beam was initially set to *E*_0_ = 264 mJ/cm^2^. Then, the input energy fluence (*E*) to the PES was decreased by placing additional neutral density filters at the exit of laser beam; *E* was reduced by 2-dB steps from −3 dB to −59 dB. Based on these conditions, *E* was calculated by *n* = 10log*E*/*E*_0_ where *n* is the attenuation by neutral density filters with a unit of dB, resulting in a range of 132 mJ/cm^2^–157 nJ/cm^2^. The input laser fluence calculated in this manner is given in Figure 3b. The incident pulsed laser energy fluence was then measured first by using the optical energy meter operated by heat accumulation. In this case, the minimum-measurable laser energy fluence was only 119.42 µJ/cm^2^ (denoted with the red arrow). Below this value, the energy fluence could not be accurately measured due to intrinsic thermal noise, resulting in signal fluctuations in time. However, the PES system allowed further measurement of signal amplitudes even down to 156.96 nJ/cm^2^. This corresponds to 1/760 of the minimum-measurable limit of the reference energy meter.

Such high-sensitivity in the PES was achieved by (1) efficient photoacoustic conversion from the CNT-PDMS transmitter and then (2) sensitive acquisition of the acoustic signal by the piezoelectric receiver with a wide aperture. We note that the photoacoustic conversion process in the CNT-PDMS composite film was previously reported with high efficiency [8], that is, a ratio of time-averaged pressure amplitude to time-averaged optical intensity; 1.4 × 10^−3^ Pa/(W/m^2^). This is due to high optical absorption and instantaneous heating efficiently utilized for thermoacoustic generation. The value was two orders of magnitude higher than those of metallic coatings. Moreover, the PES system provides an output acoustic waveform that is greatly useful in terms of quantification with peak, integration and frequency spectrum, while typical energy meters only provide an absolute amplitude without the temporal detail of short-pulse optical input.

In the measurement shown in Figure 3, the upper and lower limits lead to a wide measurable dynamic range of 55 dB for nanosecond pulsed laser irradiation. While the lower limit is determined by the noise level, the upper limit of the PES measurement is determined by the damage threshold of the CNT-PDMS transmitter against laser-induced ablation. It was previously reported that the composite structure of CNT and PDMS can withstand high-energy pulsed laser irradiation (>400 mJ/cm^2^) [4]. A well-mixed composite of CNT and PDMS coated on a glass or silica substrate allows unusual robustness for short-pulse laser irradiation, exhibiting a 7–8-fold higher damage threshold than other light-absorbers such as thin metallic films (Cr or Au) or nanostructured films consisting of an array of regular 2-D Au particles [15]. Here, we used the PES system with pulsed laser irradiation up to 132.66 mJ/cm^2^. This value was several dB lower than the damage threshold reported previously. However, our measurements conducted within this limit guarantee safe use and reliable performance. Note that spatial profiles of Nd:YAG laser beams are often non-uniform, while energy fluences are measured in an averaged manner over the entire spot. For such non-uniform beams, there can be unpredictably high optical fluence in some areas, causing damage to the CNT-PDMS transmitter.

**Figure 4:**
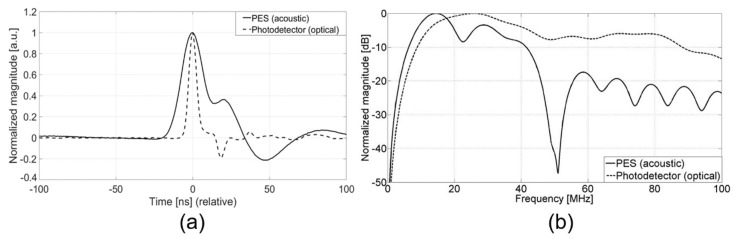
(**a**) Acoustic and optical waveforms measured by PES and high-speed photodetector. Both waveforms were normalized for pulse-width comparison. (**b**) Frequency spectra for the time-domain waveforms shown in (**a**).

Taking the advantage of the acoustic measurement possible with the PES system, we measured the input optical pulse as a time-domain waveform determined by the acoustic frequency bandwidth. Figure 4a shows the photoacoustic waveform measured by the PES for *E* = 11.8 mJ/cm^2^ which is compared with the optical pulse waveform directly measured by using a high-speed photodetector (125-MHz bandwidth). Obviously, the short laser pulse was more exactly measured by the photodetector than by the PES which produces a broader pulse shape. Here, the reference detector was a photodiode with a small active area of 0.00785 mm^2^ and a low damage threshold of 0.995 mJ/cm^2^. Such a photodiode is appropriate for high-speed temporal characterization but only with a low range of optical fluence. In contrast, the PES can be used for a high level of optical fluence, providing temporal waveforms. The previous reference energy meter used for Figure 3b may allow the measurement of high-energy optical fluence. However, in this case, temporal characterization would be hardly available for a nanosecond range of pulses due to the slow heat deposition process.

For pulse-width characterization, we used the full width at half maximum (FWHM) as a criterion for determining temporal width. Figure 4a shows that the PES waveform has a broader FWHM (21 ns) than the reference optical waveform (5.6 ns). The pulse broadening effect in the PES is due to (1) the limited frequency bandwidth of the acoustic receiver and (2) the thick depth of optical absorption in the CNT-PDMS transmitter. The bandwidth of the PVDF hydrophone used here was ~20 MHz (6-dB roll-off point). Acoustic receivers with broad bandwidth would shape short pulse waveforms more correctly. For the CNT-PDMS film, the entire thickness of optical absorption works as a spatially spread transmitter for photoacoustic generation [2]. Thus, the thicker the absorption depth, the broader the output pulse width. Thinner CNT-PDMS transmitters can be fabricated and used to reduce such broadening in the measured pulse shape [1].

Figure 4b compares the frequency spectra of two waveforms obtained by taking the Fourier transform. The transformation was performed by truncating the time-domain waveforms from −20 to 68 ns. The acoustic waveform measured by the PES had a center frequency of ~15 MHz and 6-dB widths defined at 8 and 20.6 MHz for the lower and higher edges, respectively. Due to the broadband characteristics of the reference photodiode, the optical waveform exhibited a higher center frequency of ~26 MHz and 6-dB points at 11.2 and 44.2 MHz. Although the photodiode showed better frequency characteristics than the PES, the measurable upper limit of optical fluence was greatly limited to <1 mJ/cm^2^.

**Figure 5:**
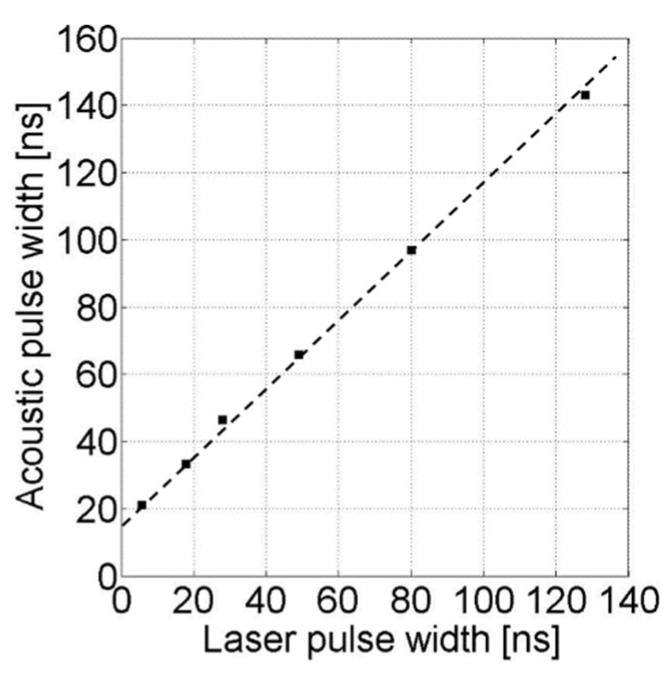
Acoustic pulse widths measured by PES for incident laser pulse widths from 6 to 130 ns.

Although the acoustic receiver used in the current PES has such limited frequency bandwidth that causes pulse broadening in time domain, we could still find the relationship between temporal widths measured optically and acoustically (Figure 5). For a fixed CNT-PDMS transmitter placed in the PES system, the output acoustic pulse width should increase with that of the incident laser pulse. As shown in Figure 5, various optical pulse widths from 6 to 130 ns were given from the Q-switched Nd:YAG laser system. The dotted line shows a linear relationship between the incident optical pulse width measured by the high-speed photodetector and the output pulse width measured by the PES. The offset in the vertical axis (15.4 ns) is due to the pulse broadening effect in the PES that is caused by the fixed optical absorption thickness within the CNT-PDMS transmitter and the limited frequency bandwidth of the acoustic receiver. By subtracting this offset, we could exactly determine the laser pulse width over 6 to 130 ns. The offset may be further reduced by using a CNT-PDMS transmitter with a thinner CNT layer than the one used here. A CNT population more densely packed onto the substrate reduces the temporal broadening, making the measured acoustic pulse width close to the optical one [1]. However, this is not necessary because the incident laser pulse width can be retrieved as long as the thickness of CNT-PDMS transmitter in the PES is fixed. This demonstrates that the PES can be used for precise measurements of nanosecond optical pulse widths.

Our PES system was utilized for the measurement of high-energy nanosecond optical pulses, using an Nd:YAG laser with a 532-nm wavelength as an input. We note that CNT has a wide absorption spectrum from visible light through THz-frequency electromagnetic waves. Thus, it can be extensively applied to pulsed THz waves or other short-pulse laser beams, for example, those with a 1064-nm wavelength. Moreover, due to such wide spectral coverage, the PES can be possibly used to measure the optical fluence of short-pulse white-light flash lamps, filled with hydrogen or nitrogen, which are available for characterization of time-resolved fluorescence. For pulse measurement shorter than a nanosecond width, PES should be improved in the following way. As sub-nanosecond pulses correspond to a frequency regime higher than hundreds of MHz, an optimal acoustic receiver should have responses to such high-frequency regime. Minimal pulse broadening in a photoacoustic transmitter would be also effective to obtain high-frequency acoustic outputs, resulting in an increased peak pressure amplitude.

We proposed PES systems that can be flexibly designed with various combinations of transmitters and acoustic receivers. Particularly, the wide-aperture acoustic receiver is suitable to fully cover an incident optical field a few cm in scale. This enables us to easily achieve high sensitivity by using a cost-effective large-area piezoelectric transducer. Our approach can be contrasted with the previous combination used for THZ wave measurement consisting of a CNT-PDMS transmitter based on a thick CNT forest and a polymer microring resonator [14]. In this configuration, the ring diameter was only 60 μm (waveguide width: a few μm) while the incident THz field had a focused spot diameter of 0.8 mm, generating the same spatial extent of pressure wave from the CNT-PDMS film. Although the optical microring resonator worked as a sensitive detector with a wide frequency bandwidth of >100 MHz, it responded to only a tiny part of the entire incident THz field (0.075%), losing most of the optical input in the detection process.

In addition to the large areal coverage of field reception available with the PES, the use of such a large area PES is important to reduce thermal load on the CNT-PDMS film. If an optical beam with 0.8 mm in diameter (the spot dimension of focused THz source [14]) is incident on the PES, the PES can measure only 2 mJ/pulse as an upper limit. However, with an acoustic receiver with a 25-mm diameter, the measurable upper limit is estimated to be as large as ~2 J/pulse for nanosecond optical pulses. It is also known that a composite of a CNT forest and PDMS has a significantly lower damage threshold than a composite with sparsely distributed CNT strands in a PDMS matrix [4]. The latter structure corresponds to our CNT-PDMS transmitter, readily allowing higher damage threshold than is the case with a dense CNT forest.

Our PES system has been used to measure nanosecond laser pulses with a low repetition rate (10 Hz). However, there are optical sources commercially available with a higher pulse repetition rate (>kHz) together with a short temporal pulse width. The PES can be possibly applied to characterize short pulses from these sources with significant sensitivity due to the highly efficient energy conversion in the CNT-PDMS transmitter and the acoustic sensing process by the piezoelectric receiver. With such high pulse repetition rates, pulse width characterization may be further improved by using additional frequency measurement algorithms [16,17].

## 4. Concluding Remarks

We fabricated a CNT-PDMS photoacoustic transmitter and combined it with a piezoelectric transducer to detect nanosecond laser pulses. The PES system could be used to measure a pulse energy as low as 156.96 nJ/cm^2^ with great reliability (up to 132.66 mJ/cm^2^). Such high sensitivity was achieved by choosing a wide-aperture acoustic receiver that can fully receive an incident optical field over a scale of mm to cm. The PES allowed the measurement of high-energy optical fluence greater than 100 mJ/cm^2^ due to the extraordinary mechanical robustness of the CNT-PDMS composite film against laser-induced damage. Moreover, unlike the commercial pyroelectric energy meter, our system could measure not only laser energy but also temporal pulse width in real time. We demonstrated a linear relationship between input optical and output acoustic pulse widths. Subtracting the offset (15.4 ns) given by the inherent pulse broadening in the PES, we could exactly retrieve the incident laser pulse width from 6 to 130 ns. We believe that our PES system can be further applied for a wide range of electromagnetic pulse energy measurement, taking advantages of its temporal reliability, accuracy, geometrical flexibility and spectral coverage over other wavelengths.
